# Role of Endogenous GLP-1 and GIP in Beta Cell Compensatory Responses to Insulin Resistance and Cellular Stress

**DOI:** 10.1371/journal.pone.0101005

**Published:** 2014-06-26

**Authors:** Srividya Vasu, R. Charlotte Moffett, Bernard Thorens, Peter R. Flatt

**Affiliations:** 1 SAAD Centre for Pharmacy and Diabetes, University of Ulster, Coleraine, Northern Ireland; 2 Centre for Integrative Genomics, University of Lausanne, Lausanne, Switzerland; University of Ulster, United Kingdom

## Abstract

Role of GLP-1 and GIP in beta cell compensatory responses to beta cell attack and insulin resistance were examined in C57BL/6 mice lacking functional receptors for GLP-1 and GIP. Mice were treated with multiple low dose streptozotocin or hydrocortisone. Islet parameters were assessed by immunohistochemistry and hormone measurements were determined by specific enzyme linked immunoassays. Wild-type streptozotocin controls exhibited severe diabetes, irregularly shaped islets with lymphocytic infiltration, decreased Ki67/TUNEL ratio with decreased beta cell and increased alpha cell areas. GLP-1 and GIP were co-expressed with glucagon and numbers of alpha cells mainly expressing GLP-1 were increased. In contrast, hydrocortisone treatment and induction of insulin resistance increased islet numbers and area, with enhanced beta cell replication, elevated mass of beta and alpha cells, together with co-expression of GLP-1 and GIP with glucagon in islets. The metabolic responses to streptozotocin in GLP-1RKO and GIPRKO mice were broadly similar to C57BL/6 controls, although decreases in islet numbers and size were more severe. In contrast, both groups of mice lacking functional incretin receptors displayed substantially impaired islet adaptations to insulin resistance induced by hydrocortisone, including marked curtailment of expansion of islet area, beta cell mass and islet number. Our observations cannot be explained by simple changes in circulating incretin concentrations, suggesting that intra-islet GLP-1 and GIP make a significant contribution to islet adaptation, particularly expansion of beta cell mass and compensatory islet compensation to hydrocortisone and insulin resistance.

## Introduction

Glucagon-like peptide-1 (GLP-1) together with the sister incretin hormone gastric inhibitory polypeptide (GIP) are secreted from intestinal L-cell and K-cells in response to feeding and exert pleiotropic metabolic effects [1]–[5]. Notably GLP-1 and GIP exert key actions on islets, including stimulation of insulin release, augmentation of glucose sensitivity, promotion of beta cell replication and protection from both beta-cell apoptosis and cytotoxic attack. Important and emerging actions are also evident at extrapancreatic sites [1], [5]. Apart from positive actions on cognition via effects at the hippocampus, the effects of the two peptides differ, with GIP inhibiting gastric acid secretion and exerting anabolic effects on both adipose tissue and bone whereas GLP-1 inhibits gastric emptying and satiety, while affording cardioprotection [5]–[8]. The antidiabetic effects of GLP-1 receptor activation have been harnessed for type 2 diabetes with the development of stable GLP-1 mimetics as a new class of therapeutic drugs [9], [10]. DPPIV inhibitors which normally block the rapid enzymatic breakdown of both incretin hormones and additionally augment the actions of GIP have also been introduced [11].

Intestinal L-cells and pancreatic islet alpha cells both express the proglucagon gene which is differentially processed in a tissue specific manner, yielding GLP-1 in the gut and glucagon in the islets. The classical view is that in islet alpha cells, proglucagon is processed primarily by PC2 to generate the 29 amino acid sequence of glucagon plus glicentin-related pancreatic polypeptide (GRPP) and major proglucagon fragment, whereas the proglucagon precursor is processed by PC1/3 in intestinal L-cells to generate GLP-1 together with GLP-2, glicentin, oxyntomodulin and GRPP [3]. However, recent observations challenge this view, showing that islet alpha cells express PC1/3 and produce significant amounts of GLP-1 [12]–[15] which has been confirmed in human islets using MALDI-TOF mass spectrometry as GLP-1(7–36) and GLP-1(7–37) [16]. Further, alpha cell expression of GLP-1 is enhanced under situations of increased insulin demand and beta cell stress such as glucose toxicity, chemical insult and partial pancreatectomy [14], [15], [17]–[19]. Consistent with this view, *in vitro* studies indicate that isolated islets secrete GLP-1 upon alpha cell stimulation with arginine and that chemical blockade of GLP-1 action suppresses concomitant insulin release [20].

Although less well studied, bioactive GIP is also synthesized and released by islet alpha-cells [21]. Co-localisation of GIP and glucagon has been demonstrated using highly specific antibodies in alpha cells of many species, including Burmese phython, rat and human [21]. Studies by the Vancouver group have also demonstrated GIP messenger RNA in islets and confirmed that bioactive GIP is secreted by islets in response to arginine stimulation [21]. It appears that both GIP (1–42) and GIP (1–30) are expressed due to processing of proGIP by PC1/3 and PC2. Indeed, the major peptide form in alpha-cells might be GIP (1–30) due to the relative abundance of PC2 under normal circumstances and its ability to mediate a second C-terminal cleavage of GIP (1–42) liberating the truncated peptide [22]. These two forms have identical biological effects at the GIP receptor, including promotion of insulin secretion and lowering of blood glucose [23].

Although significant evidence for islet alpha cell production of GLP-1 and GIP has been gathered which suggests a biological role [14], [15], [16], [18], [21], [24]–[28], there is no real evidence that this plays any part in the regulation of islet function. It is unlikely that islet-derived GLP-1 and GIP contribute significantly to circulating incretin concentrations or the extra-pancreatic actions of the peptides, but locally released GLP-1 and GIP might exert important effects on neighboring islet cells. Thus, on the basis of known actions of the incretins [9], [5], [29], it can be hypothesized that local islet production of incretin peptides is likely to enhance beta-cell function and survival in response to cytotoxic attack and increased demand imposed by insulin resistance.

In this paper, we have used incretin receptor knock-out mice and wild-type controls to evaluate the role of islet and intestinal L- and K-cell derived GLP-1 and GIP in relation to alterations in number, morphology and function of the islets and beta-cells in animal models of beta cell insult and insulin resistance, induced by multiple low dose streptozotocin or hydrocortisone treatment. The results provide novel information on the regulation of beta cell mass, functional consequences of intra-islet expression of incretin hormones and add weight to the debate concerning strategies for exploitation of incretin receptors in relation to obesity-diabetes [30].

## Materials and Methods

### Animals

Adult 12 week old male wild-type C57BL/6 mice and both GLP-1RKO and GIPRKO mice on the same C57BL/6 genetic background were bred in house at the Biomedical and Behavioural Research Unit (BBRU) at University of Ulster, Coleraine. The original background of these incretin receptor knockout mice are described elsewhere [31], [32]. Mice were housed individually in an air-conditioned room at 22±2°C with 12 h light and 12 h dark cycle. Standard rodent maintained pellet diet (Trouw Nutrition, Northwich, UK) and drinking water were available *ad libitum*. All animal experiments were carried out in accordance with the UK Animals (Scientific Procedures) Act 1986 and approved by the University of Ulster Animal Ethics Review Committee. All necessary steps were taken to ameliorate any potential animal suffering and animals were sacrificed by lethal inhalation of CO2 followed by cervical dislocation.

### *In vivo* studies

Over a 5-day period, incretin receptor knockout mice and age-matched controls (n = 6 in each group, fasted for 4 h) received once daily i.p. injections (13∶00 h) of saline vehicle (0.9% (w/v), NaCl) or streptozotocin (50 mg/kg body wt; made freshly in 0.1 M sodium citrate buffer, pH 4.5). Food intake, body weight and blood glucose were monitored daily. Mice were culled at 5 days post treatment to procure pancreata and terminal blood. In a second series of experiments, incretin receptor knockout mice and controls (n = 6) received once daily i.p. injections (09∶00 h) of saline vehicle (0.9% (w/v), NaCl) or hydrocortisone (70 mg/kg body wt; made freshly in saline) for 10 days. Food intake, body weight and blood glucose were monitored daily. Mice were culled at end of hydrocortisone treatment to procure pancreata and terminal blood. Pancreata were halved longitudinally for histology and hormone measurements.

### Biochemical analyses

Pancreatic tissues were homogenised and extracted using buffer containing 20 mM Tris HCl (pH 7.5), 150 mM NaCl, 1 mM EDTA, 1 mM EGTA and 0.5% Triton X 100 and stored at −80°C. Protein concentrations were determined using Bradford reagent (Sigma, Dorset, UK). Insulin in plasma and pancreatic tissue extracts was determined by radioimmunoassay [33]. Total GLP-1 and GIP in plasma and pancreatic extracts were determined using specific enzyme linked immunoassays, following manufacturer’s instructions (GLP-1 Total ELISA, EZGLP-1T-36K, Millipore; rat/mouse GIP ELISA, EZRMGIP-55K, Millipore). Glucagon in plasma and pancreatic tissue extracts was determined using glucagon chemiluminescent assay (EZGLU-30K, Millipore), following manufacturer’s instructions.

### Tissue processing for histological analyses

Pancreatic tissues were fixed in 4% paraformaldehyde for 48 h at 4°C and then processed using automated tissue processor (Leica TP1020, Leica Microsystems, Nussloch, Germany). After embedding in paraffin wax, tissues were sectioned at 7 µm thickness using a microtome (Shandon finesse 325, Thermo scientific, UK) and sections were picked at an interval of 10 sections. The tissue sections were deparaffinised using Histoclear II (National Diagnostics, UK) and rehydrated through series of ethanol. After antigen retrieval at 94°C for 20 min using citrate buffer (pH 6.0), the sections were blocked using 10% normal goat serum and incubated with primary antibodies (Table 1) overnight at 4°C. The sections were then incubated with secondary antibody (Table 1) for 45 min at 37°C. The slides were then mounted using anti-fade mounting medium and viewed under FITC filter (488 nm) or TRITC filter (594 nm) using a fluorescent microscope (an Olympus system microscope, model BX51) and photographed using the DP70 camera adapter system. Antibodies used were highly specific and showed no cross-reactivity with related peptide hormones.

**Table 1:** Antibodies.

*Primary antibodies*				
Target	Host	Clonality	Dilution	Source
Insulin	Mouse	Monoclonal	1∶1000	Abcam, ab6995
Glucagon	Guinea-pig	Polyclonal	1∶200	Raised in-house – PCA2/4
GLP-1	Rabbit	Polyclonal	1∶200	Raised in-house – XJIC8
GIP	Rabbit	Polyclonal	1∶400	RIC34/111J, kindly donated by Professor L Morgan, Guildford, UK
Ki67	Rabbit	Polyclonal	1∶200	Abcam, ab15580
***Secondary antibodies***				
**Target**	**Host**	**Reactivity**	**Dilution**	**Source**
IgG	Goat	Guinea-pig	1∶400	Alexa Fluor 488, Invitrogen, UK
IgG	Goat	Mouse	1∶400	Alexa Fluor 594, Invitrogen, UK
IgG	Goat	Rabbit	1∶400	Alexa Fluor 488, Invitrogen, UK
IgG	Goat	Rabbit	1∶400	Alexa Fluor 594, Invitrogen, UK

### Hematoxylin & eosin staining for demonstration of lymphocytic infiltration

After rehydration using a series of ethanol solutions, the sections were exposed to hematoxylin solution for 5 min and rinsed with tap water, acid alcohol (0.25% HCl, 50% methanol) and again in tap water prior to staining with eosin for 5 min. Following rinsing with distilled water, sections were dehydrated using ethanol, dipped in histo-clear II for 2 min and mounted using DePeX mounting medium. The slides were then viewed using Olympus IX51 inverted microscope and photographed using a SPOT RT-Ke camera (Diagnostic Instruments Inc, Sterling Heights, MI).

### Image analysis

Cell∧F image analysis software (Olympus Soft Imaging Solutions, GmbH) was used to analyse islet parameters including islet area, alpha cell area and beta cell area, expressed as µm^2^. Number of islets per mm^2^ of pancreas and islets with central alpha cells were determined in a blinded fashion. For analysis of islet size distribution, islets smaller than 10,000 µm^2^ were designated ‘small’, greater than 10,000 µm^2^ and lesser than 25,000 µm^2^ were designated ‘medium’ and greater than 25,000 µm^2^ were designated ‘large’. Ki67 and TUNEL positive, insulin positive cells were counted in a blinded manner and expressed as beta cell proliferation or apoptosis frequency (% of total beta cells analysed). Approximately 2000 beta cells were counted for Ki67 and TUNEL analysis. Balance between proliferation and apoptosis was expressed as ratio of proliferation to apoptosis frequencies. Alpha cells containing GLP-1/GIP and negligible amounts of glucagon (red or orange staining) were counted and expressed as GLP-1 positive alpha cells/islet (% of total number of alpha cells). For scoring insulitis [34], a grading system was used in which grade 0 refers to no infiltration, grade 1 refers to peri-vascular or peri-ductular infiltration, grade 2 refers to peri-islet infiltration and intra-islet infiltration of less than 50% of islet and grade 3 refers to intra-islet infiltration of more than 50% of islet. For blinded observations, the investigator was unaware of the treatment groups and codes were disclosed after statistical analyses.

### Statistical analysis

Results are expressed as mean ± S.E.M, n = 6 mice in each group. Data were compared using One-way ANOVA, followed by a Bonferroni *Post hoc* test using GraphPad PRISM software. Area under the curve (AUC) analyses was calculated using the trapezoidal rule with baseline subtraction. *p*<0.05 was considered to be statistically significant.

## Results

### Streptozotocin and hydrocortisone significantly alter islet parameters

**Figure 1:**
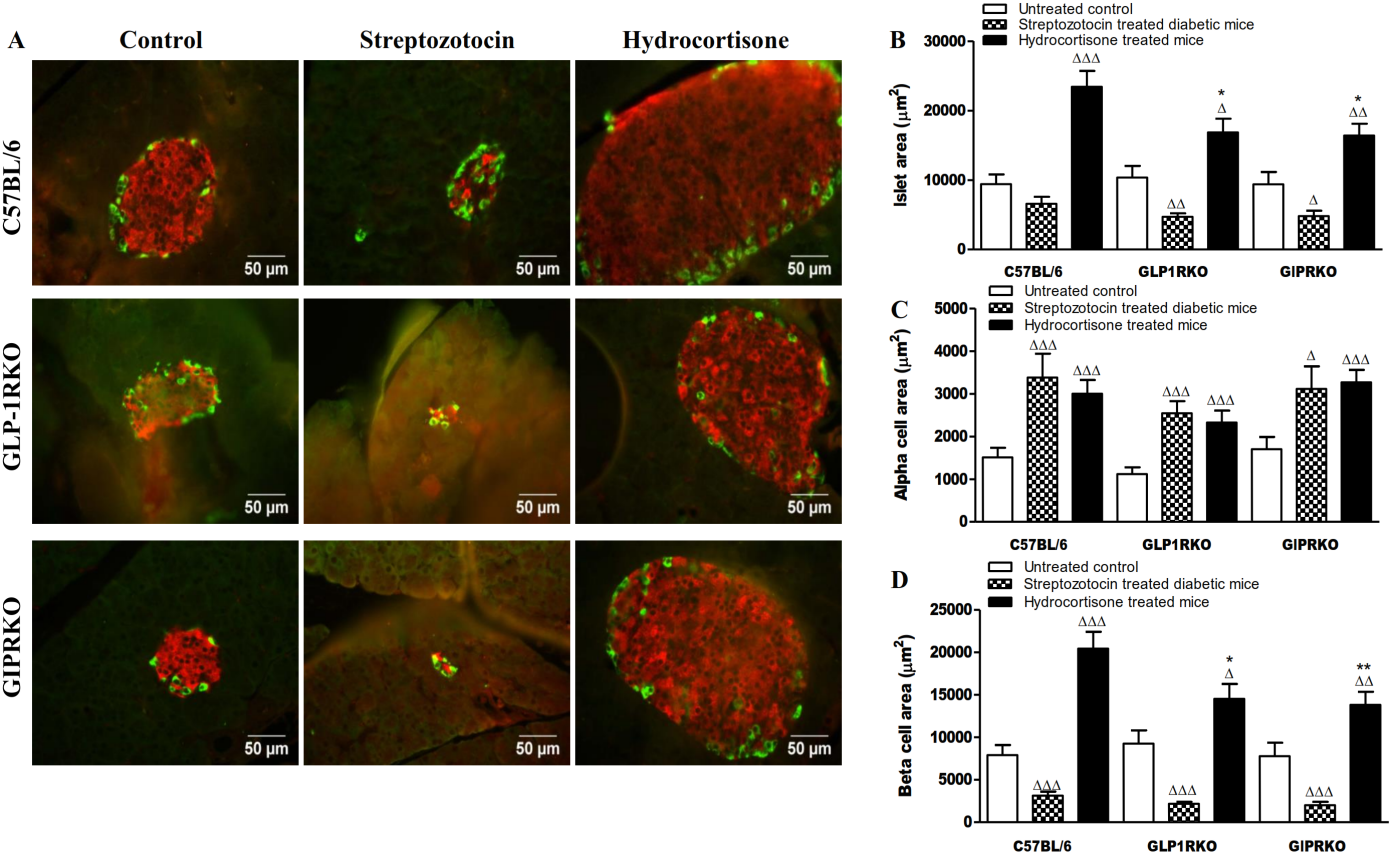
Islet analysis. A Representative islets showing insulin (red) and glucagon (green) immunoreactivity from C57BL/6, GLP-1RKO and GIPRKO mice. B – Islet area (µm^2^). C - Alpha cell area (µm^2^). D – Beta cell area (µm^2^). Values are mean ± SEM (n = 6).^ Δ^p<0.05, ^ΔΔ^p<0.01, ^ΔΔΔ^p<0.001 compared to respective controls. *p<0.05, **p<0.01 compared to respective treatment of C57BL/6 mice.

**Figure 2:**
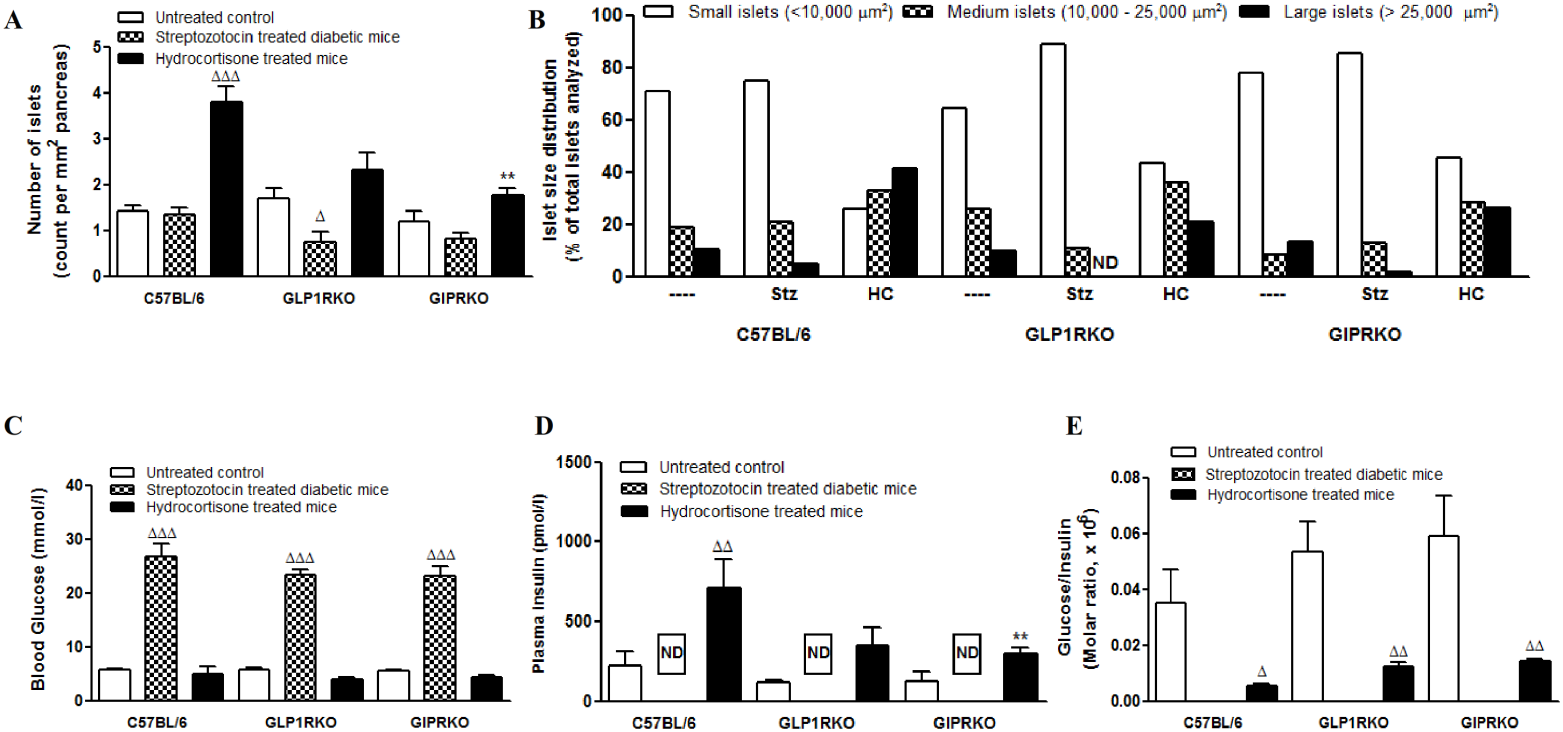
Islet analysis. A – Number of islets (count per mm^2^ of pancreas). B – Islet size distribution (% of total islets analysed). C – Blood glucose (mmol/l). D – Plasma insulin (pmol/l). E – Glucose/Insulin molar ratio. Values are mean ± SEM (n = 6). ^Δ^p<0.05, ^ΔΔ^p<0.01, ^ΔΔΔ^p<0.001 compared to respective controls. **p<0.01 compared to respective treatment of C57BL/6 mice.

Representative images showing insulin (red) and glucagon (green) staining in islets of control, streptozotocin and hydrocortisone treated C57BL/6, GLP-1RKO and GIPRKO mice are shown in Figure 1A. In C57BL/6 mice, streptozotocin reduced islet area, although not significantly, in association with an increase in alpha-cell area and a marked reduction in beta-cell area (p<0.001, Figure 1B, C, D). In incretin receptor KO mice, streptozotocin significantly reduced islet area (p<0.05, p<0.01, Figure 1B), with broadly similar increases in alpha-cell area (p<0.05, p<0.001, Figure 1C), and decreases in beta-cell area as wild-type mice (p<0.001, Figure 1D). In contrast to these effects of streptozotocin, hydrocortisone significantly increased islet area, alpha and beta cell areas in C57BL/6 mice (p<0.05, p<0.01, p<0.001, Figure 1B, C, D). However, in incretin receptor KO mice, hydrocortisone-induced increases in islet and beta cell areas were significantly lower than wild-type controls (p<0.05, p<0.01, Figure 1B, D). Hydrocortisone also markedly increased number of islets per mm^2^ of pancreas in C57BL/6 mice but not in receptor KO mice (p<0.001, Figure 2A). Indeed the number of islets in hydrocortisone treated GIPRKO mice was less than C57BL/6 mice (p<0.01, Figure 2A). Hydrocortisone decreased numbers of small islets and increased large islets in C57BL/6 mice with the opposite being observed in both groups of KO mice (Figure 2B). Streptozotocin significantly decreased number of islets in GLP-1RKO mice but not in other groups (p<0.05, Figure 2A). Streptozotocin had relatively little effect on size distribution with tendency for decreased percentage of larger islets (Figure 2B).

**Figure 3:**
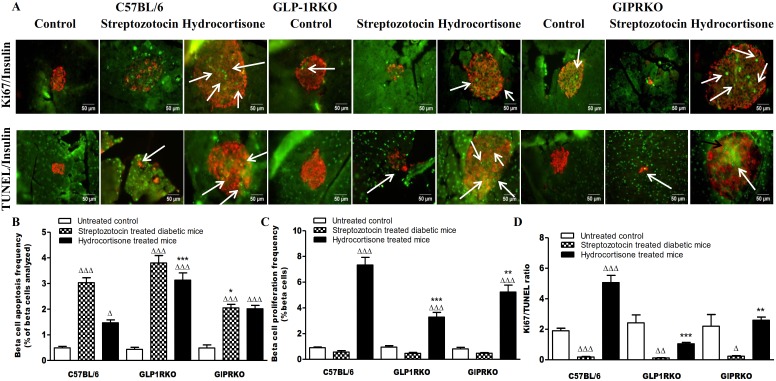
Proliferation and apoptosis. A Representative islets showing insulin (red) and Ki67 (green, indicated by arrows), insulin (red) and TUNEL (green, indicated by arrows) immunoreactivity from C57BL/6, GLP-1RKO and GIPRKO mice. B – Beta cell apoptosis frequency (% of beta cells analysed). C – Beta cell proliferation frequency (% of beta cells analysed). D – Ki67/TUNEL ratio. Values are mean ± SEM of 6 observations unless otherwise indicated. ^Δ^p<0.05, ^ΔΔ^p<0.01, ^ΔΔΔ^p<0.001 compared to respective controls. *p<0.05, **p<0.01, ***p<0.001 compared to respective treatment of C57BL/6 mice.

Representative islets showing Ki67/insulin and TUNEL/insulin staining in islets of control, streptozotocin and hydrocortisone treated C57BL/6, GLP-1RKO and GIPRKO mice are shown in Figure 3A. Streptozotocin and hydrocortisone markedly increased beta cell apoptosis frequency in all groups (p<0.05, p<0.001, Figure 3B). Hydrocortisone significantly increased beta cell proliferation frequency in all groups (p<0.001, Figure 3B). However, in KO mice, proliferation frequency was significantly lesser than C57BL/6 mice (p<0.01, p<0.001, Figure 3C). Streptozotocin significantly decreased Ki67/TUNEL ration in all groups, thus favoring beta cell apoptosis (p<0.05, p<0.01, p<0.001, Figure 3D). Hydrocortisone significantly increased Ki67/TUNEL ratio in C57BL/6 but did not alter Ki67/TUNEL ratio in KO mice (p<0.001, Figure 3D). Ki67/TUNEL ratio was markedly lower in hydrocortisone treated KO mice when compared to C57BL/6 mice (p<0.01, p<0.001, Figure 3D).

**Figure 4:**
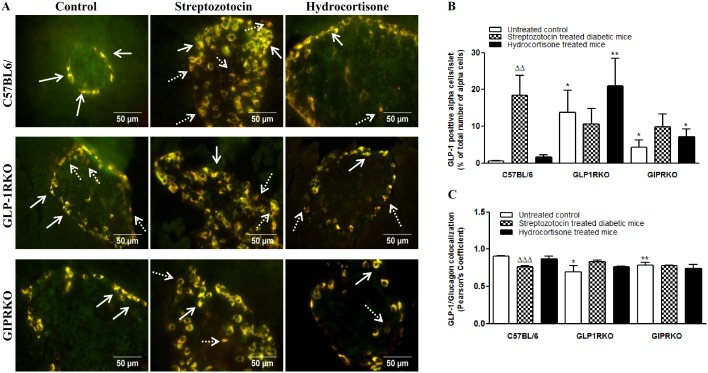
Glucagon and GLP-1 colocalization. A Representative islets showing glucagon (green) and GLP-1 (red) immunoreactivity from C57BL/6, GLP-1RKO and GIPRKO mice. Dotted arrows indicate alpha cells expressing mainly of GLP-1 while block arrows indicate colocalization with glucagon (yellow). B – Islets with GLP-1 positive alpha cells (% of total number of alpha cells analysed). C – GLP-1/Glucagon colocalization (Pearson’s colocalization coefficient). Values are mean ± SEM of 6 observations unless otherwise indicated. ^ΔΔ^p<0.01, ^ΔΔΔ^p<0.001 compared to respective controls. *p<0.05, **p<0.01 compared to respective treatment of C57BL/6 mice.

Representative images of the co-localization of glucagon with GLP-1 in islets of control, streptozotocin and hydrocortisone treated C57BL/6, GLP-1RKO and GIPRKO mice are shown in Figure 4A. GLP-1 immunoreactivity was detectable in alpha cells of all untreated control groups. Streptozotocin significantly increased GLP-1 positive cells per islet while decreasing colocalization with glucagon (p<0.01, p<0.001, Figure 4B, C). In untreated incretin receptor KO mice, GLP-1 positive alpha cells were more numerous and colocalization with glucagon was less than in C57BL/6 mice (p<0.05, Figure 4B, C). GLP-1 positive alpha cells in hydrocortisone treated incretin receptor KO mice were increased compared with C57BL/6 mice (p<0.05, p<0.01, Figure 4B).

**Figure 5:**
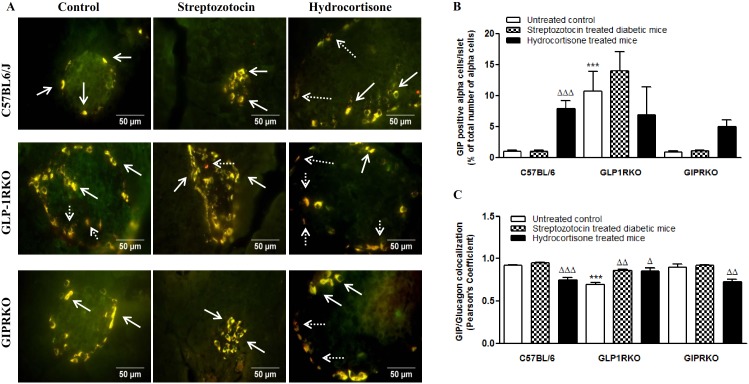
Glucagon and GIP colocalization. A Representative islets showing glucagon (green) and GIP (red) immunoreactivity from C57BL/6, GLP-1RKO and GIPRKO mice. Dotted arrows indicate alpha cells expressing mainly of GIP while block arrows indicate colocalization with glucagon (yellow). B – GIP positive alpha cells (% of total number of alpha cells analysed). C – GIP/glucagon colocalization (Pearson’s coefficient of colocalization). Values are mean ± SEM of 6 observations. ^Δ^p<0.05, ^ΔΔ^p<0.01, ^ΔΔΔ^p<0.001 compared to respective non-pregnant controls. ***p<0.001 compared to respective treatment of C57BL/6 mice.

Representative images showing the co-localization of glucagon with GIP in islets of all groups are shown in Figure 5A. GIP positive alpha cells in hydrocortisone treated C57BL/6 mice were more numerous than saline treated controls, while colocalization with glucagon was less (p<0.001, Figure 5B, C). In GLP-1RKO mice, streptozotocin and hydrocortisone did not alter GIP positive alpha cells but significantly increased colocalization (p<0.05, p<0.01, Figure 5C). There was a tendency towards increase in GIP positive alpha cells in hydrocortisone treated GIPRKO mice while colocalization with glucagon was decreased significantly (p<0.01, Figure 5C). In untreated GLP-1RKO mice, GIP positive alpha cells were more numerous and colocalization with glucagon was less than in C57BL/6 mice (p<0.001, Figure 5B, C).

### Streptozotocin induces lymphocyte infiltration in islets

**Figure 6:**
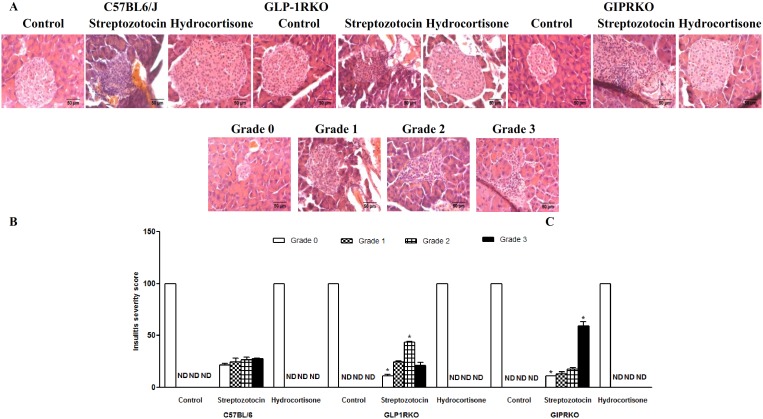
Lymphocyte infiltration. A Representative islets showing hematoxylin and eosin staining of islets from C57BL/6, GLP-1RKO and GIPRKO mice. B –Insulitis severity score (% of total number of islets analysed and graded according to level of lymphocyte infiltration). Values are mean ± SEM of 6 observations. *p<0.05 compared to respective treatment of C57BL/6 mice.

Representative images showing hematoxylin and eosin staining in islets of the various groups of mice are shown in Figure 6A. Multiple low dose streptozotocin induced lymphocyte infiltration in islets of all groups. The degree of infiltration was determined by blinded analysis of insulitis grading [34]. Control and hydrocortisone treated mice showed no signs of lymphocyte infiltration (Figure 6B). In streptozotocin treated incretin receptor KO mice, islets without insulitis (grade 0) were significantly less numerous than C57BL/6 mice. Grade 2 and grade 3 insulitis were significantly higher in GLP-1RKO and in GIPRKO mice, respectively compared with streptozotocin treated C57BL/6 mice (p<0.05, Figure 6C).

### Streptozotocin and hydrocortisone alter pancreatic and plasma hormone contents

Streptozotocin significantly increased blood glucose levels in all groups of mice (p<0.001, Figure 2C) while insulin was consistently undetectable in the circulation following streptozotocin treatment (Figure 2D). Hydrocortisone did not significantly affect blood glucose levels in any of the groups (Figure 2C). However it significantly increased circulating levels of insulin in C57BL/6 mice but not in incretin receptor knockout mice (p<0.01, Figure 2D). Plasma insulin was significantly increased compared with hydrocortisone treated GIPRKO mice (p<0.01, Figure 2D). Consistent with induction of insulin resistance, hydrocortisone treatment significantly lowered glucose to insulin molar ratio compared to untreated controls in all groups (p<0.05, p<0.01, Figure 2E).

**Figure 7:**
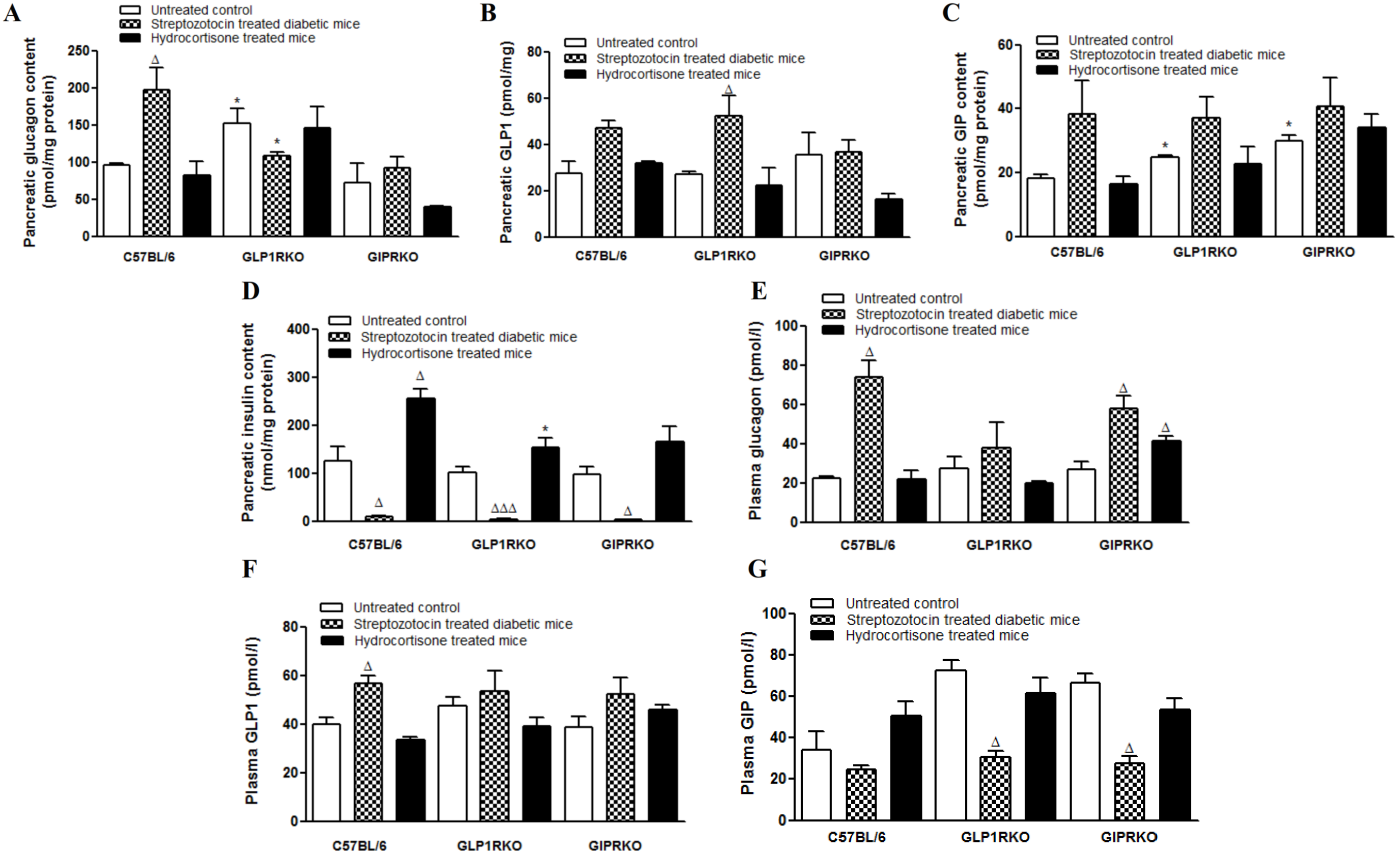
Pancreatic and plasma levels of hormones. A – Pancreatic glucagon content (pmol/mg protein). B – Pancreatic GLP-1 content (pmol/mg protein). C – Pancreatic GIP content (pmol/mg protein). D – Pancreatic insulin content (nmol/mg protein). E – Plasma glucagon (pmol/l). F – Plasma total GLP-1 (pmol/l). G – Plasma GIP (pmol/l). Values are mean ± SEM of 4–6 observations unless otherwise indicated. ^Δ^p<0.05, ^ΔΔΔ^p<0.001 compared to respective controls. *p<0.05 compared to respective treatment of C57BL/6 mice.

Streptozotocin increased pancreatic glucagon content in C57BL/6, with no effect in other groups (p<0.05, Figure 7A). Pancreatic glucagon content in streptozotocin treated GLP-1RKO mice was significantly less than C57BL/6 mice (p<0.05, Figure 7A). Hydrocortisone did not affect pancreatic glucagon content in any of the groups. Streptozotocin increased pancreatic GLP-1 and GIP content although not significantly in C57BL/6 mice (Figure 7B, C). The increase of pancreatic GLP-1 was significant in streptozotocin treated GLP-1RKO mice (p<0.05, Figure 7B). Hydrocortisone did not affect pancreatic GLP-1 or GIP content in any of the groups (Figure 7 B, C). In untreated incretin receptor knockout mice, pancreatic GIP content was significantly higher than untreated C57BL/6 mice (p<0.05, Figure 7C).

Streptozotocin significantly lowered pancreatic insulin content in all groups of mice (p<0.05, p<0.001, Figure 7D) while hydrocortisone increased pancreatic insulin content only in C57BL/6 mice (p<0.05, Figure 7D). After hydrocortisone treatment, pancreatic insulin content of C57BL/6 mice was significantly more than GLP-1RKO mice (p<0.05, Figure 7D). Streptozotocin significantly increased circulating levels of glucagon in C57BL/6 and GIPRKO mice (p<0.05, Figure 7E). Whereas hydrocortisone did not affect circulating glucagon in C57BL/6 and GLP-1RKO mice (Figure 7E), it significantly increased plasma glucagon in GIPRKO mice (p<0.05, Figure 7F). Streptozotocin increased plasma GLP-1 in C57BL/6 mice while it decreased plasma GIP in both groups of incretin receptor knockout mice (p<0.05, Figure 7H).

## Discussion

Regulation of beta-cell function is under the control of circulating nutrients, hormones, paracrine interactions and autonomic nerves innervating the pancreatic islets [35]. Although classically considered as enteroinsular hormones [36], this study has examined the possibility that intra-islet production of GLP-1 or GIP, together with circulating incretins from the gut, contributes to mechanisms controlling beta-cell function, particularly the regulation of beta-cell mass and adaptive responses to beta cell toxins and insulin resistance. As with other recent studies, we readily demonstrated GLP-1 and GIP in glucagon-containing islet alpha-cells by immunohistochemistry using antisera specific for glucagon or the two incretin hormones [14]–[16], [18], [19], [21], [24]–[28]. Comparison of molar quantities measured by ELISA revealed approximately similar amounts of GLP-1 and GIP in the pancreas, corresponding to levels approximately 25% of pancreatic glucagon. When released from within islets [20], [21], local concentrations of GIP and particularly GLP-1 are likely to be much greater than the low circulating levels of the hormones and without significant exposure to degradation by DPPIV [37].

Administration of multiple low doses of streptozotocin resulted in severe insulitis, marked destruction of beta-cells, depletion of pancreatic and plasma insulin, elevation of glucagon and severe diabetes. This is consistent with the observed actions of the toxin to induce lymphocytic infiltration and insulitis rather than simply inducing chemical destruction of beta cells as occurs when administered as a large single dose [38]. The marked loss of beta cells resulted in remaining islets being marginally smaller, irregularly shaped and exhibiting substantially increased alpha-cell mass and pancreatic glucagon content. Interestingly, a subpopulation of alpha cells predominantly expressed GLP-1, supporting the idea that an increase in local production of GLP-1 may be involved in compensation for beta cell loss. There was also a substantial increase in TUNEL staining beta cells and, in contrast to other reports [39], little evidence of Ki-67 positive beta-cell regeneration, giving rise to markedly decreased Ki-67 to TUNEL ratio.

The possible involvement of GLP-1 and GIP in this compensatory islet response was evaluated further using GLP-1R KO and GIPR KO mice maintained on the C57BL/6 background. Compared with wild-type C57BL/6 controls, incretin receptor KO mice displayed characteristic changes of islet morphology and beta-cell mass as described previously [40]. GLP-1 and GIP were expressed in islet alpha cells of all groups of mice. Treatment of incretin receptor KO mice with multiple low dose streptozotocin resulted in a slightly greater severity of islet damage than normal mice treated with the toxin. This was reflected by decreased islet numbers in GLP-1R KO mice and decreases in islet size and beta cell area together with increased alpha cell mass in both transgenic mouse models. Knock-out of GLP-1R also resulted in increased pancreatic GLP-1 without change of pancreatic glucagon or circulating hormone levels, suggesting that insulin deficiency and/or receptor deletion affects cellular GLP-1 production from the proglucagon precursor. In contrast, circulating GIP was decreased in in both groups of receptor knock-out mice without affecting tissue stores. Irrespective of these changes, deletion of receptors for either incretin hormone did not greatly affect the course of severe insulin deficient diabetes. This likely reflects the severe hyperglycaemia and substantial level of damage inflicted on beta cells, as witnessed by lack of beta cell regeneration and markedly decreased Ki67/TUNEL ratios in control diabetic C57BL/6 mice.

The metabolic and islet cell responses to hydrocortisone treatment were quite different to those induced by multiple low doses of streptozotocin. Thus, C57BL/6 animals exhibited markedly increased islet, beta and alpha cell areas associated with increased numbers of medium and large sized islets. Ki67 proliferation was enhanced and there was only a small level of apoptosis as judged by TUNEL staining. Both pancreatic and plasma levels of insulin but not glucagon were markedly elevated but glucose concentrations were relatively normal, indicating the effectiveness of markedly enhanced beta cell activity to overcome severe insulin resistance [41]. In addition to such metabolic actions on islets, we cannot discount that hydrocortisone might also exert direct effects on the function of beta and alpha cells [42]. Intra-islet expression of GLP-1 and GIP was clearly evident with increased numbers of alpha cells mainly producing GIP. Pancreatic and circulating levels of GLP-1 and GIP were unchanged, thereby excluding a significant role of intestinally-derived hormones in cellular and metabolic effects. Thus, ablation of intra-islet, as opposed to circulating GLP-1 or GIP, actions using receptor KO mice appear to be responsible for loss of the normal compensatory increases of islet numbers and morphology induced by hydrocortisone, with islet size and beta cell mass remarkably less than observed in normal C57BL/6 mice. Numbers of islets in GIPR KO mice were also less than hydrocortisone treated C57BL/6 mice. Accordingly, it appears that the sole or dual additive actions of GLP-1 and GIP produced locally by alpha cells may be particularly important in terms of islet responses to increased functional demand.

These combined observations suggest that GLP-1 and GIP produced largely by islet alpha cells may play a hitherto unproven role in islet adaptation to insulin resistance and the control of glucose homeostasis. Further studies using mice with specific knock-out of incretin receptors in islets would be useful to investigate this further. Decreased beta cell mass and inability to secrete appropriate amounts of insulin are classical features of gestational as well as type 1 and type 2 diabetes [43], [44]. Since existing therapies target insulin replacement or enhancing insulin secretion/action [45], intra-islet production of GLP-1 and GIP through manipulation of proconvertase enzymes may represent a therapeutically useful way to increase beta cell mass and physiological insulin secretion.
